# Comprehensive investigation of the clinical significance of long non-coding RNA HOXA-AS2 in acute myeloid leukemia using genome-wide RNA sequencing dataset

**DOI:** 10.7150/jca.48045

**Published:** 2021-02-21

**Authors:** Rui Huang, Xiwen Liao, Xiangkun Wang, Qiaochuan Li

**Affiliations:** 1 Department of Hematology, The First Affiliated Hospital of Guangxi Medical University, Nanning, 530021, Guangxi Zhuang Autonomous Region, People's Republic of China.; 2 Department of Hepatobiliary Surgery, The First Affiliated Hospital of Guangxi Medical University, Nanning, 530021, Guangxi Zhuang Autonomous Region, People's Republic of China.

**Keywords:** HOXA-AS2, acute myeloid leukemia, The Cancer Genome Atlas, molecular mechanism, drug prediction

## Abstract

**Objective:** The present study aimed to determine the prognostic value of HOXA cluster antisense RNA2 (HOXA-AS2) in acute myeloid leukemia (AML), and to explore its potential molecular mechanisms. We also screening of potential drugs targeting HOXA-AS2 in AML.

**Methods:** The level 3 raw genome-wide RNA sequencing dataset of AML was download from The Cancer Genome Atlas (TCGA) Data Portal, and the potential molecular mechanisms and drugs prediction of HOXA-AS2 in AML were explored using multiple bioinformatics analysis approaches.

**Results:** TCGA AML cohort dataset indicated that HOXA-AS2 was significantly up-regulated in AML bone marrow tissues, and high HOXA-AS2 expression was related to poor overall survival (log-rank *P*=0.0284, hazard ratio 1.640, 95% confidence interval 1.046-2.573). Functional enrichment of differentially expressed genes (DEGs) suggested that the difference in prognosis between AML patients with high- and low-HOXA-AS2 expression may be due to differences in biological processes and pathways, including cell adhesion, angiogenesis, mitogen-activated protein kinase, cell differentiation, and other biological processes, and phosphatidylinositol 3 kinase-protein kinase B and Wnt signaling pathways. We also screened out three potential HOXA-AS2-targeted therapeutic drugs for AML, megestrol, carmustine, and cefoxitin, based on these DEGs. Functional enrichment analysis of HOXA-AS2-co-expressed genes revealed that HOXA-AS2 may act a part in AML by regulating nuclear factor-κB transcription factor activity, DNA methylation, angiogenesis, apoptosis, cell migration, Toll-like receptor 4, and Wnt signaling pathways.

**Conclusion:** Our findings suggest that HOXA-AS2 is up-regulated in the bone marrow in patients with AML, and may serve as a novel prognostic biomarker for AML.

## Introduction

Acute myeloid leukemia (AML) is a clonal malignant disease of the hematopoietic system 1, 2. The etiology of AML is closely related to genetic factors, and genetic variation plays an important role in its diagnosis, classification, risk prediction, precision treatment, and prognosis prediction 3-5. The recent application of immunology, cytogenetics, molecular biology, and other technologies, has led to a deeper understanding of the biological characteristics of tumor cells in AML, which has in turn laid the foundations for the accurate classification, diagnosis, and prognostic prediction in AML, as well as for the selection of optimal treatment methods 6, 7.

Long non-coding RNAs (lncRNAs) play an important role in many biological activities, such as dose-compensation, epigenetic regulation, cell cycle regulation, and cell differentiation regulation, and are also associated with many diseases, including cancers and AML. LncRNA mainly functions via its secondary structure and binds to proteins, resulting in chromatin remodeling and affecting transcription factor function. In addition, lncRNAs can also bind to microRNAs, indirectly affecting mRNA expression, or can bind directly to mRNAs, affecting mRNA translation, shearing, and degradation processes. Numerous studies have shown that the lncRNA HOXA-AS2 plays an oncogenic role in a variety of cancers 8. Dong and his coworkers demonstrated that HOXA-AS2 played a indispensable role in the resistance of AML cells to adriamycin and could thus be a potential target for adriamycin resistance in AML patients 9. The Cancer Genome Atlas (TCGA) is an open access database containing a large number of tumor multi-omics high-throughput sequencing datasets, including AML 10. The aim of the present study is to identify the prognostic value of HOXA-AS2 in AML, and to explore its potential molecular mechanisms using a TCGA genome-wide RNA sequencing dataset. We also aimed to screening of potential HOXA-AS2-targeted drugs in AML.

## Materials and methods

### Data acquisition

The level 3 raw RNA sequencing (RNA-seq) dataset of AML bone marrow tissues and the clinical prognostic parameters for 151 patients with AML were got from TCGA (https://portal.gdc.cancer.gov/) 11, including mRNA and lncRNA expression datasets. *EdgeR* was used to normalized the RNA-seq dataset 12. After matching the RNA-seq data with the clinical prognostic parameters, 130 AML patients with both RNA-seq dataset and clinical prognostic parameters were finally included for subsequent analysis. Of these, 21 AML patients were excluded, including 10 with a survival time of 0, and 11 patients with no survival information.

### Expression distribution and prognostic value of HOXA-AS2 expression in AML

Box plots of HOXA-AS2 expression between bone marrow tissues of AML patients and healthy subjects were derived from the Gene Expression Profiling Interactive Analysis (GEPIA) website (http://gepia.cancer-pku.cn/index.html), based on the TCGA dataset and the GTEx (http://www.gtexportal.org/home/) projects 13. Patients were divided into high- and low-HOXA-AS2-expression groups by the median expression value, and overall survival (OS) was compared between the two groups. The accuracy of HOXA-AS2 for predicting OS in patients with AML was evaluated using a time-dependent receiver operating characteristic (ROC) curve, and drawn by the *survivalROC* package (https://cran.r-project.org/web/packages/survivalROC/index.html) using the R platform 14, 15.

### Molecular mechanisms and drug prediction of HOXA-AS2 in AML

We further explored the molecular mechanisms underlying the prognostic differences between AML patients with different HOXA-AS2 expression levels by screening differentially expressed genes (DEGs) using the *edgeR* package. Genes that met the following criteria were considered to be DEGs: |log_2_ fold change (FC)|>1, *P* value <0.05, and false discovery rate (FDR) value <0.05. These DEGs were then subjected to functional enrichment analysis using the Database for Annotation, Visualization, and Integrated Discovery v6.8 (DAVID v6.8, https://david.ncifcrf.gov/tools.jsp) 16, 17, Biological Networks Gene Ontology tool (BiNGO) 18, and Gene Set Enrichment Analysis (GSEA) 19. We also examined the gene co-expression interactions of these DEGs in bone marrow tissues of AML patients using the Weighted Gene Co-Expression Network Analysis (WGCNA) method, as described previously 20. The meaning of degree in the network is connectivity. The protein-protein interaction (PPI) and gene-gene interaction (GGI) networks of these DEGs were also verified using The Search Tool for the Retrieval of Interacting Genes/Proteins (STRING) database (http://string.embl.de/) 21-23 and GeneMANIA (http://genemania.org/) 24, 25, respectively. We further screened potential drugs for AML patients with high HOXA-AS2 expression by Connectivity Map (CMap, https://portals.broadinstitute.org/cmap/) analysis of these DEGs 26, 27. Drugs with a mean connectivity score <-0.2 and a *P* value <0.05 were considered as potential targeted therapeutic drugs for AML patients with high HOXA-AS2 expression. We also performed a survival analysis of these DEGs.

### Functional enrichment of HOXA-AS2 in AML

LncRNAs act by regulating related protein-coding genes. Screening genes co-expressed with HOXA-AS2 in AML bone marrow tissues by enrichment analysis will thus help us to understand the molecular mechanism of HOXA-AS2 in AML. We therefore screened protein-coding genes (PCGs) co-expressed with HOXA-AS2 in the whole genome RNA-seq dataset using the* cor* function through the R platform. PCGs significantly correlated with the HOXA-AS2 expression (Pearson coefficient* P* value <0.05) were considered to be co-expressed with HOXA-AS2 in AML bone marrow tissue 28, 29. Functional enrichment analysis of the co-expressed PCGs was performed using DAVID v6.8 and BiNGO, and PPI and GGI networks were constructed using STRING and GeneMANIA, respectively. We also performed a survival analysis of these co-expressed PCGs.

### Statistical analysis

FDR in *edgeR* and GSEA were carried out according to the Benjamini-Hochberg approach 30-32. The Kaplan-Meier curves of genes in AML were calculated using the log-rank test, in addition, survival package were used for hazard ratios (HRs) and 95% confidence intervals (CIs) calculation in the R platform. A *P* value <0.05 was considered significant. SPSS version 20.0 (IBM Corporation, Armonk, NY, USA) and R 3.5.0 (https://www.r-project.org/) were used for all statistical analyses.

## Results

### Expression distribution and prognostic value of HOXA-AS2 expression in AML

**Figure 1:**
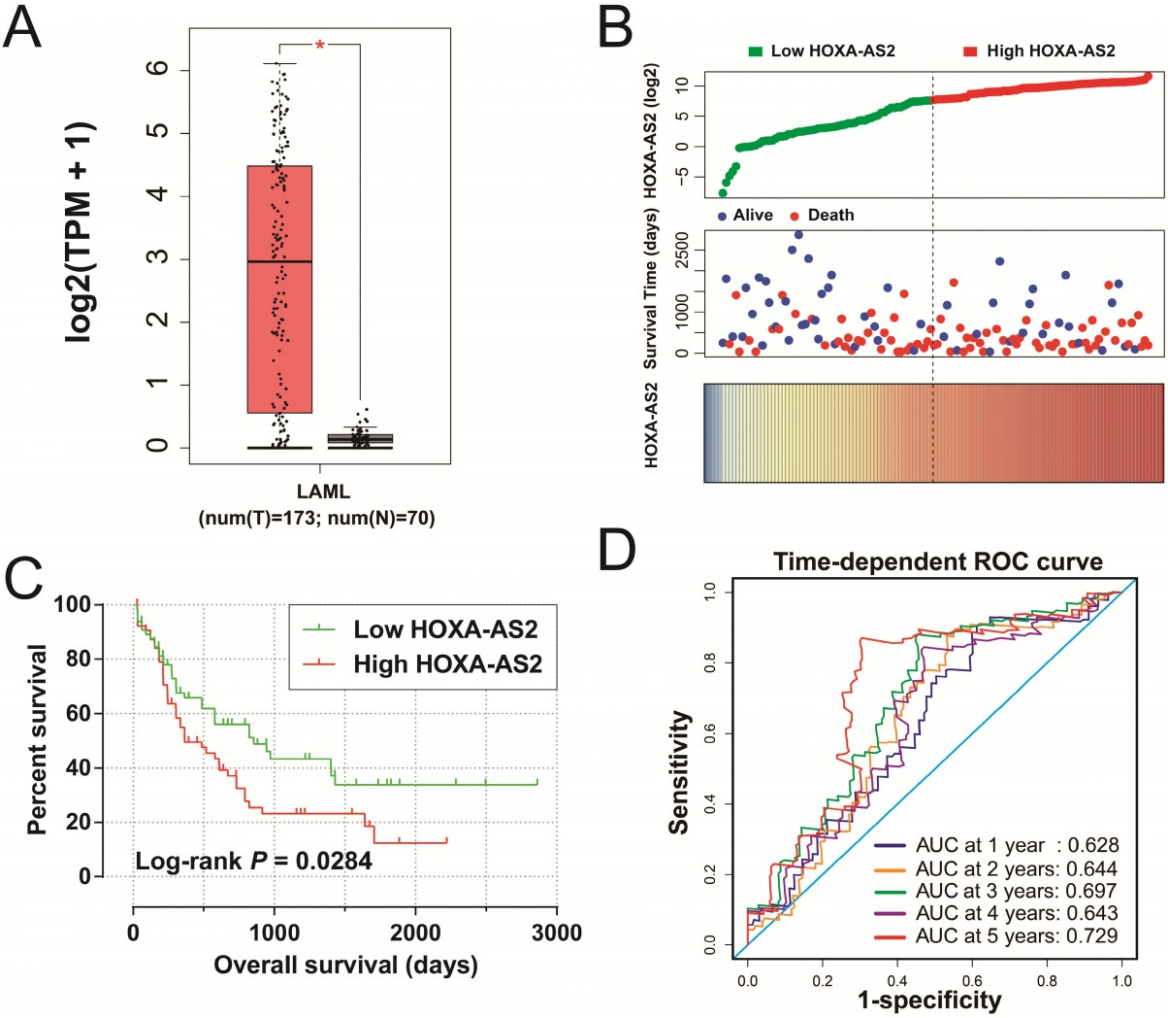
Clinical significance of HOXA-AS2 expression in AML. (A) Box plot of HOXA-AS2 expression distribution in bone marrow tissues of AML patients and healthy subjects (* *P* < 0.05). (B) Distribution map of survival time and expression of HOXA-AS2 in AML patients. (C) Kaplan-Meier curve of HOXA-AS2 in the TCGA AML cohort. (D) Time-dependent ROC curve of HOXA-AS2 expression for predicting OS in patients with AML.

We obtained box plots of HOXA-AS2 expression in bone marrow tissues of AML patients and healthy subjects using the GEPIA website, and showed that expression levels were significantly up-regulated in AML patients (**Figure 1A**). Survival analysis demonstrated that high expression of HOXA-AS2 was notably related to poorer OS in patients with AML (log-rank *P*=0.0284, HR=1.640, 95%CI=1.046-2.573) (**Figure 1B, 1C**). Time-dependent ROC curve analysis confirmed that HOXA-AS2 expression showed a high accuracy for predicting AML 5-year survival, with an area under the curve of 0.729 (**Figure 1D**).

### Molecular mechanisms of HOXA-AS2 and drug prediction in AML

**Figure 2:**
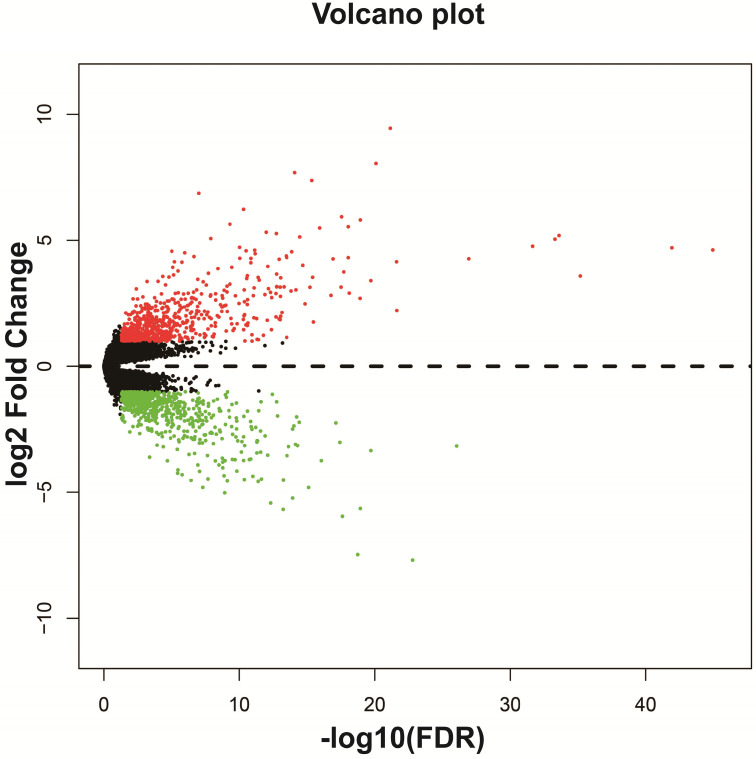
Volcano plot of DEGs in low- and high-HOXA-AS2-expressing AML patients.

We screened DEGs in a whole-genome RNA-seq dataset of bone marrow tissues from AML patients with different HOXA-AS2 expression levels using the *edgeR* package. There were 1,314 genes were considered as DEGs, including 659 down-regulated and 655 up-regulated genes (**Table S1**). A heat map of the DEGs was shown in **Figure S1**, as well as the volcano plot was shown in **Figure 2**. We subjected these DEGs to functional enrichment analysis to explore the potential mechanisms responsible for the prognostic difference between AML patients with low- and high-HOXA-AS2-expression levels. Gene ontology (GO) term analysis performed by DAVID v6.8 indicated that these DEGs were prominently related to cell adhesion, cell-cell signaling, cell migration involved in sprouting angiogenesis, single organismal cell-cell adhesion, positive regulation of mitogen-activated protein kinase (MAPK) activity, cell differentiation, positive regulation of extracellular regulated protein kinase (ERK) 1 and ERK2 cascade, integrin-mediated signaling pathway, positive regulation of MAPK cascade, cytokine receptor activity, and the epoxygenase P450 pathway (**Table S2**). Kyoto Encyclopedia of Genes and Genomes (KEGG) enrichment analysis suggested that these DEGs were prominently related to extracellular matrix (ECM)-receptor interactions, phosphatidylinositol 3 kinase (PI3K)-protein kinase B (PKB/Akt) signaling pathway, focal adhesion, pathways in cancer, cell-adhesion molecules (CAMs), cytokine-cytokine receptor interactions, calcium, cyclic adenosine monophosphate (cAMP), and Wnt signaling pathways (**Table S3**). BiNGO enrichment analysis also suggested that these DEGs were prominently related to cell differentiation, cell adhesion, cell migration, angiogenesis, cell proliferation, regulation of the Notch signaling pathway, regulation of the cAMP biosynthetic process, regulation of cell growth, the integrin-mediated signaling pathway, positive regulation of cell division, and tumor necrosis factor receptor activity (**Figure S2, Table S4**).

**Figure 3:**
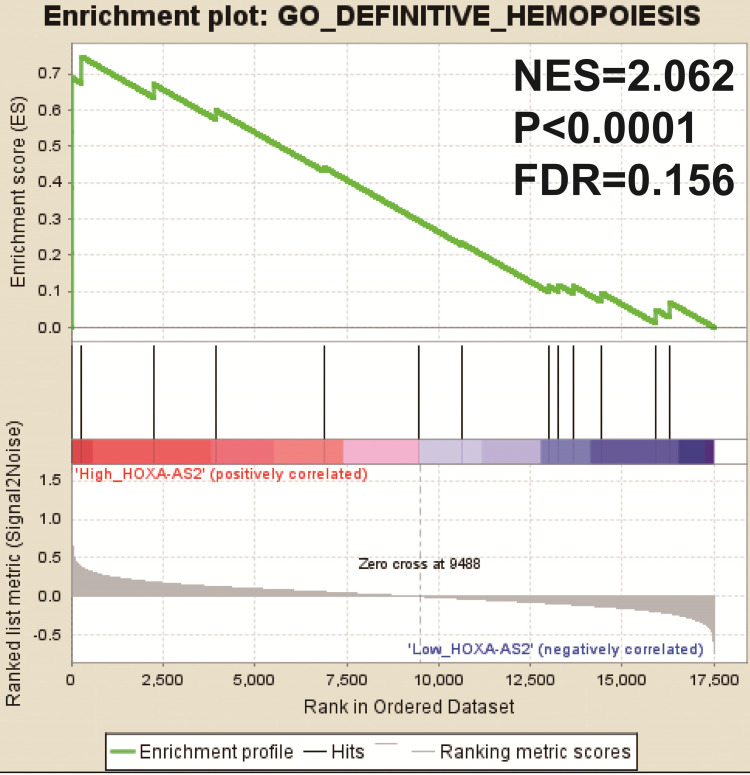
GSEA results in low- and high-HOXA-AS2-expressing AML patients using the c5 (c5.all.v6.2.symbols.gmt) reference gene set.

**Figure 4:**
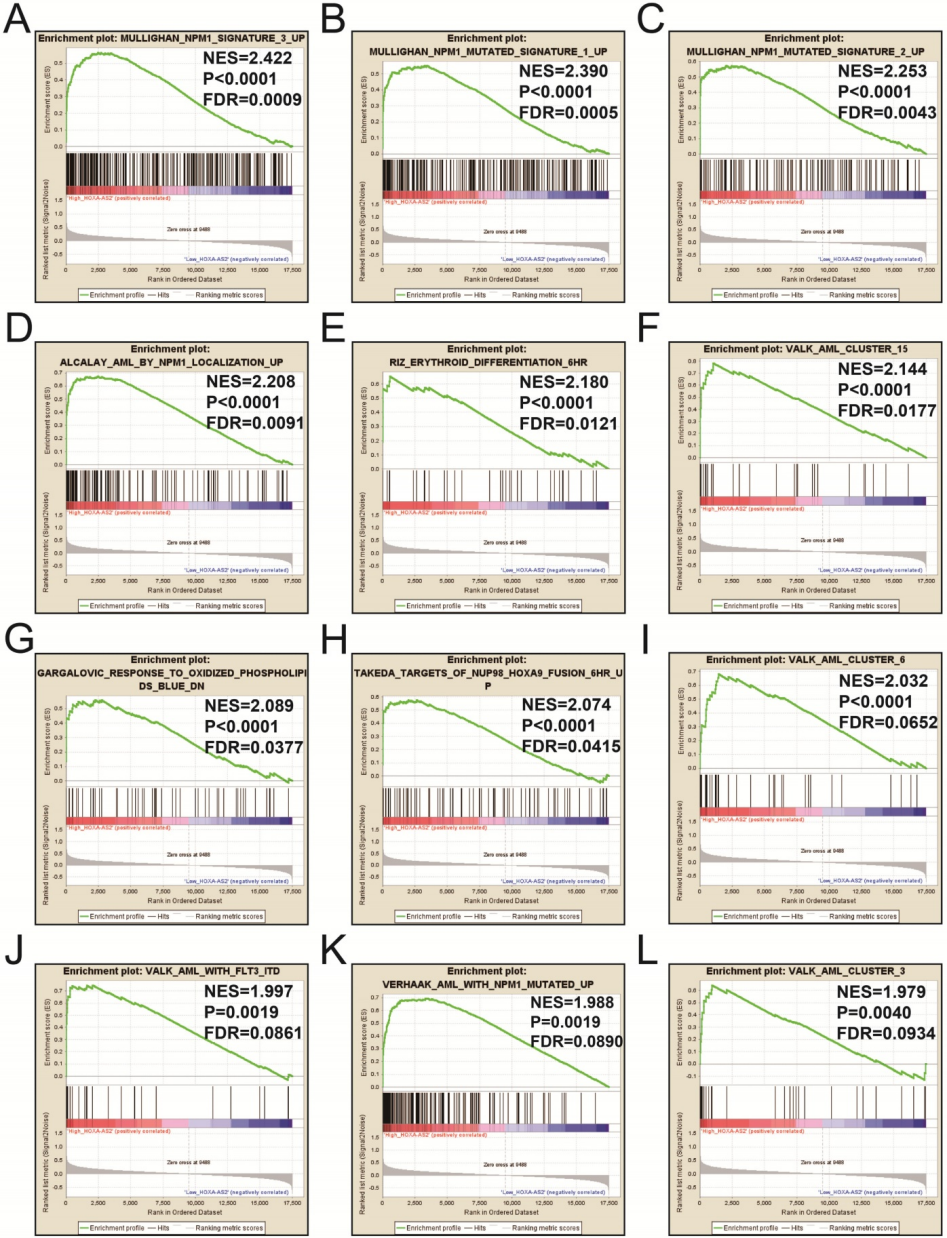
GSEA results in low- and high-HOXA-AS2-expressing AML patients using the c2 (c2.all.v6.2.symbols.gmt) reference gene set (A-L).

We also investigated the potential mechanisms responsible for the prognostic difference between low- and high-HOXA-AS2-expressing groups using the GSEA approach. GSEA analysis using the c5 (c5.all.v6.2.symbols.gmt) gene set revealed that definitive hemopoiesis biological processes may be involved in the prognostic difference between these two groups (**Figure 3**). In contrast, GSEA analysis using the c2 (c2.all.v6.2.symbols.gmt) gene set revealed gene signatures related to up-regulated in pediatric AML with mutated NPM1, AML with internal tandem duplications (IDT) in fms-related tyrosine kinase 3 (FLT3), targets of nucleoporin 98 (NUP98)-homeobox A9 (HOXA9) fusion, AML cluster, erythroid differentiation, and response to oxidized phospholipids (**Figure 4A-L**). All the above pathways enriched by GSEA were related to AML.

**Figure 5:**
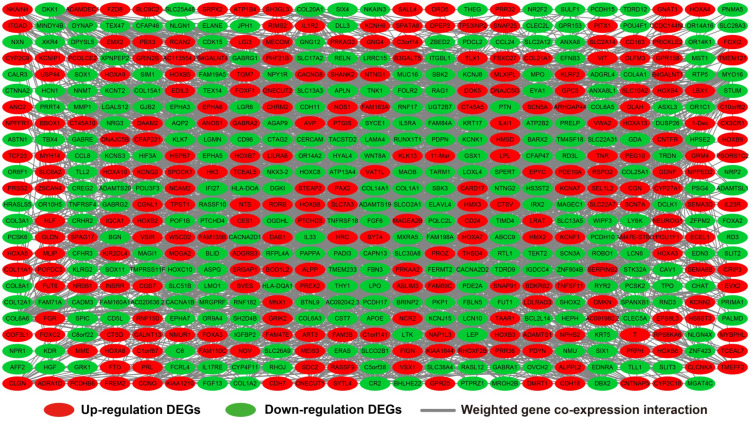
WGCNA regulatory network of DEGs.

**Figure 6:**
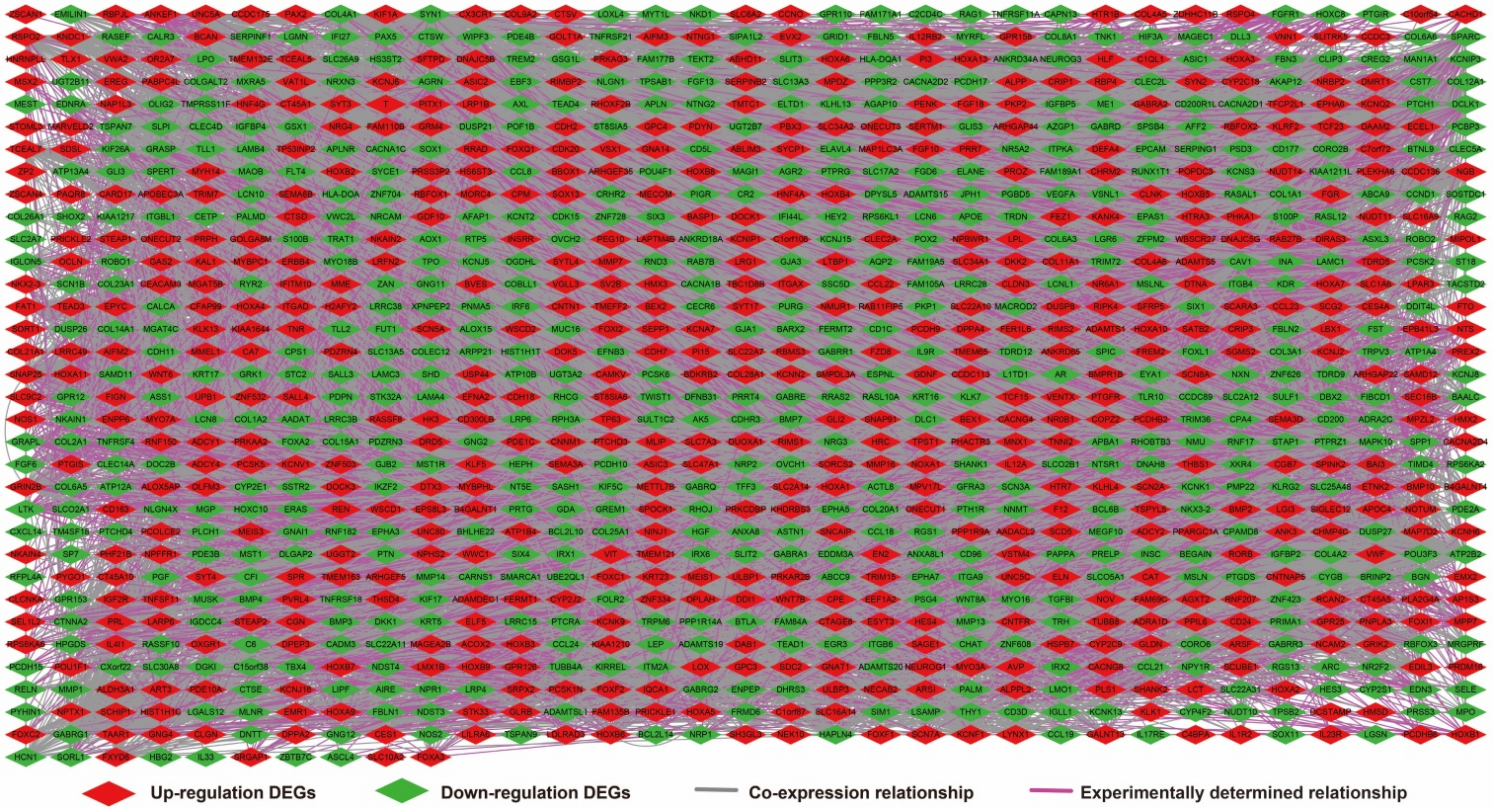
Regulatory network of DEGs constructed using the STRING online tool.

**Figure 7:**
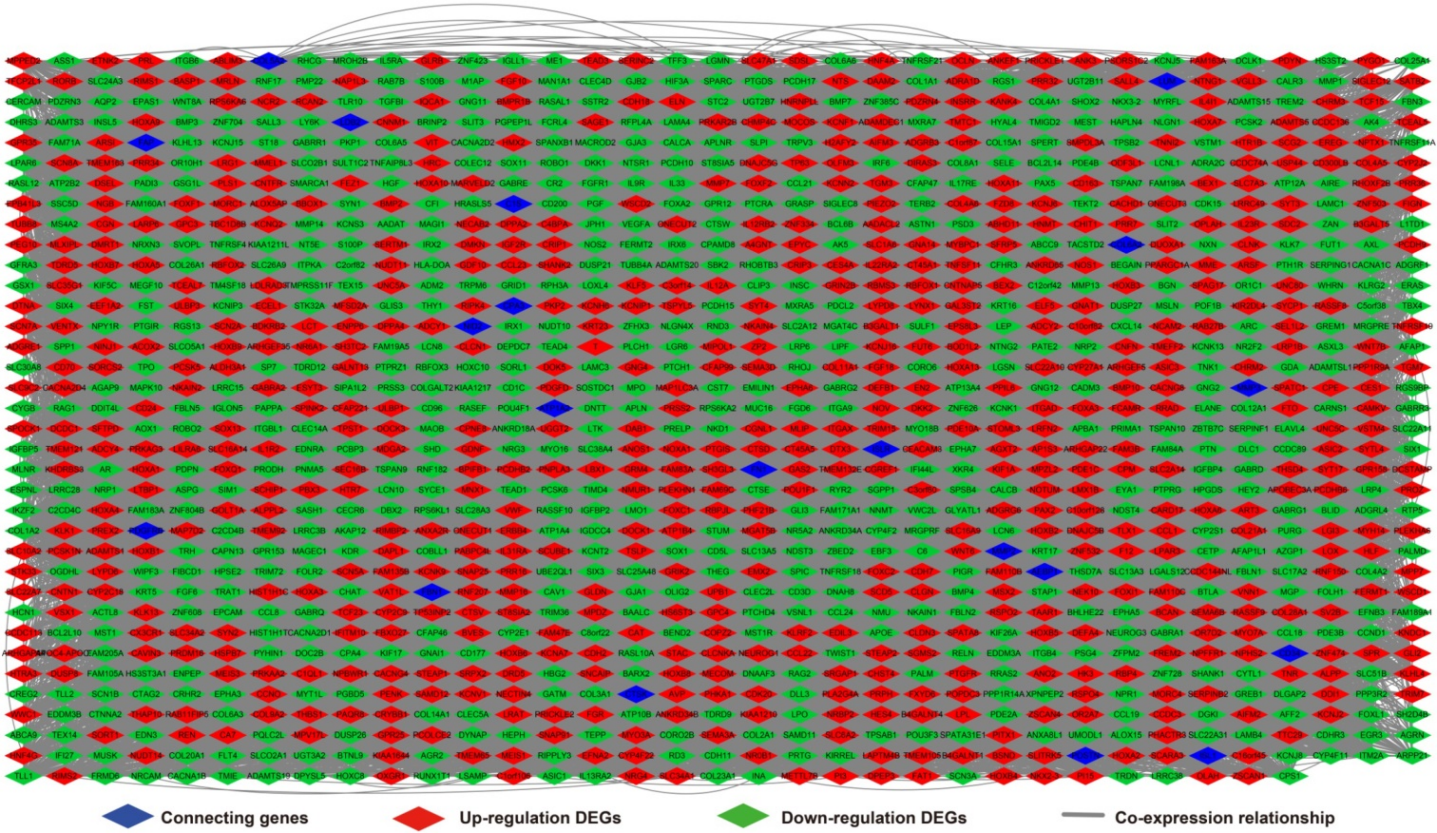
Regulatory network of DEGs constructed using the GeneMANIA online tool.

**Figure 8:**
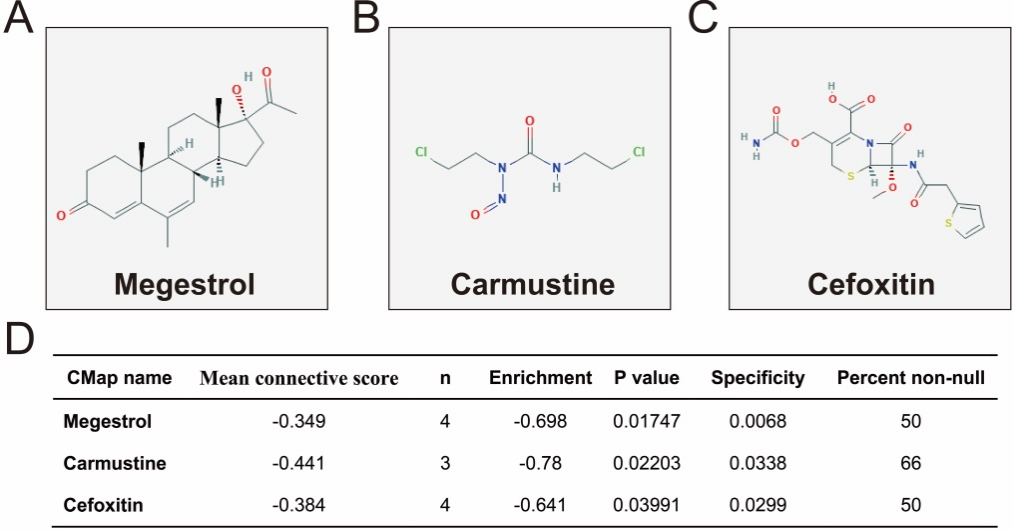
Results of CMap analysis. Chemical structures of (A) megestrol, (B) carmustine, and (C) cefoxitin. (D) Summary of CMap analysis results.

**Figure 9:**
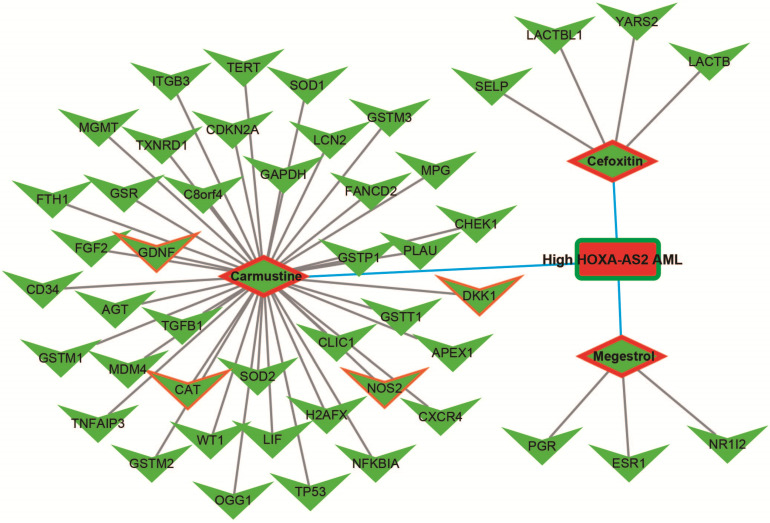
Drug-protein interaction networks constructed using the STITCH online tool.

We further investigated the interactions among these DEGs using the WGCNA, STRING, and GeneMANIA approaches to construct interaction regulatory networks (**Figures 5, 6**, and **7**, respectively). The soft threshold distribution plot and module clustering plot of WGCNA analysis are shown in **Figure S3A-D**. The highest degree DEGs in the WGCNA network were PCDH17 and RNF182, with a degree of 55. These analyses demonstrated that these DEG had complex co-expression interaction relationships. We also performed a CMap analysis to predict potential HOXA-AS2-targeted therapeutics for AML. Using CMap analysis, we screened three small-molecule drugs, megestrol, carmustine, and cefoxitin, which could be used as targeted therapy for HOXA-AS2 in AML. PubChem (https://pubchem.ncbi.nlm.nih.gov/) provides the chemical structures of these targeted drugs 33, 34 (**Figure 8A-C**) and the detailed results of the CMap analysis are summarized in **Figure 8D**. We then used the online search tool for interactions of chemicals (STITCH; http://stitch.embl.de) 35, 36 to construct drug-protein interaction networks for these three drugs (**Figure 9**). By matching the drug-related genes and DEGs, four DEGs appeared in these three drug regulatory networks. We therefore concluded that carmustine may play a role in AML patients with high HOXA-AS2 expression levels by regulating these four DEGs: glial cell derived neurotrophic factor (*GDNF*), catalase (*CAT*), nitric oxide synthase 2 (*NOS2*), and dickkopf WNT signaling pathway inhibitor 1 (*DKK1*). By analyzing the connectivities of these four genes in the WGCNA network, we found that the *DKK1* gene had a high degree of 50 in the WGCNA network, with high connectivity and regulation ability. However, the remaining three genes had lower degrees in the network, suggesting that carmustine was likely to exert its therapeutic effect in AML mainly by regulating *DKK1*.

**Figure 10:**
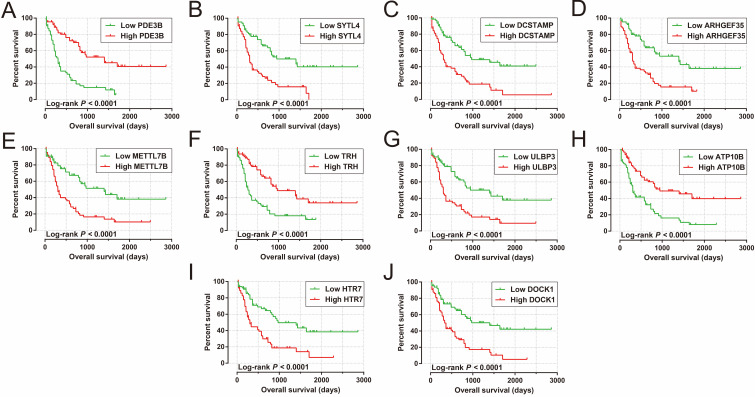
Kaplan-Meier curves of the top ten significantly prognostic DEGs. (A) *PDE3B*, (B) *SYTL4*, (C) *DCSTAMP*, (D) *ARHGEF35*, (E) *METTL7B*, (F) *TRH*, (G) *ULBP3*, (H) *ATP10B*, (I) *HTR7*, and (J) *DOCK1* in AML OS.

We also carried out a survival analysis to determine the prognostic values of these DEGs. There were 324 DEGs were prominently related to OS of AML in the present study (**Table S5**). The top ten significantly prognostic DEGs were as follows (**Figure 10A-J**): phosphodiesterase 3B (*PDE3B*), synaptotagmin-like 4 (*SYTL4*), dendrocyte-expressed seven transmembrane protein (*DCSTAMP*), Rho guanine nucleotide exchange factor 35 (*ARHGEF35*), methyltransferase-like 7B (*METTL7B*), thyrotropin-releasing hormone (*TRH*), UL16-binding protein 3 (*ULBP3*), ATPase phospholipid-transporting 10B (*ATP10B*), 5-hydroxytryptamine receptor 7 (*HTR7*), and dedicator of cytokinesis 1 (*DOCK1*).

### Functional enrichment of HOXA-AS2 in AML

**Figure 11:**
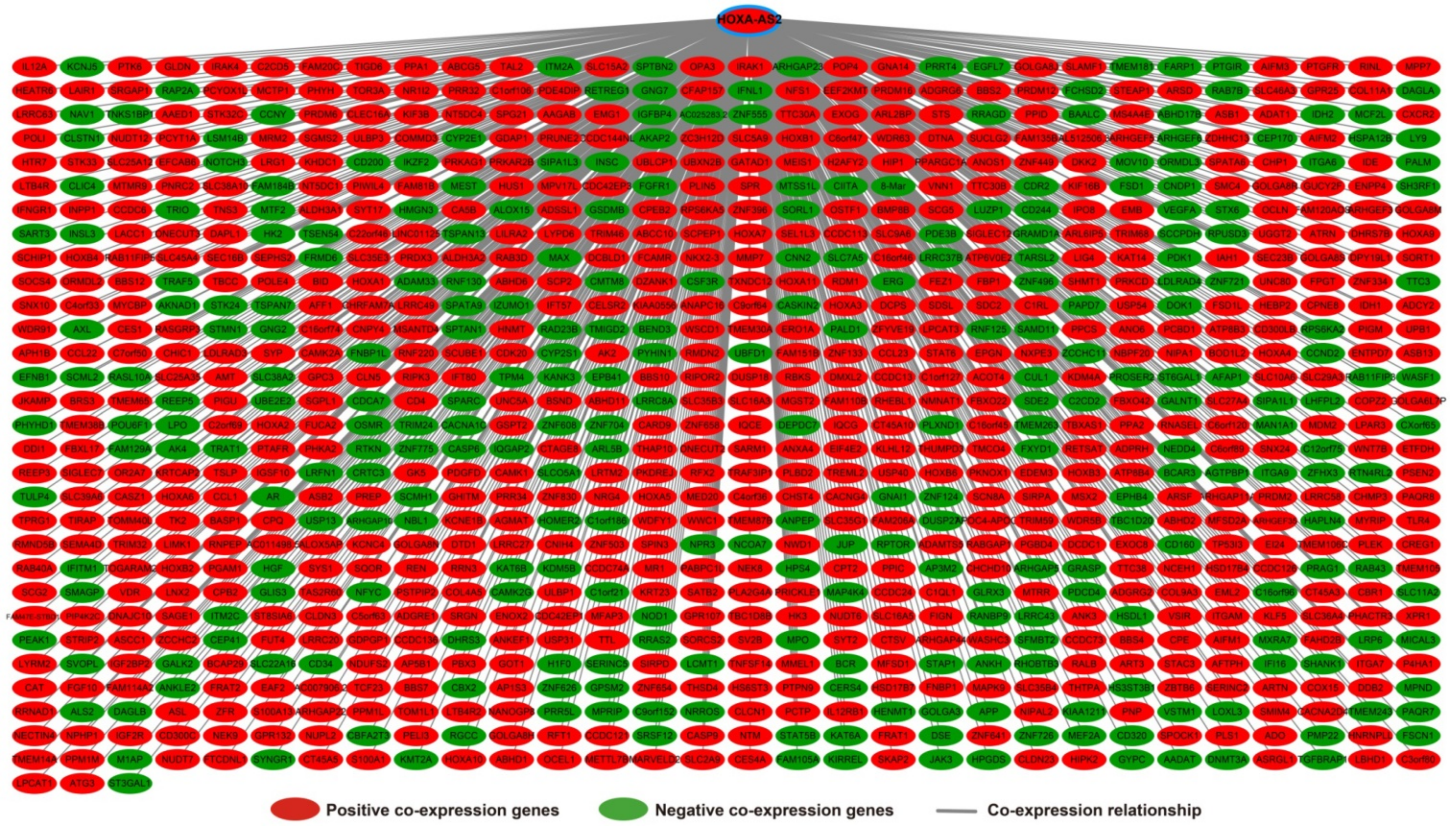
Co-expression of regulatory networks of HOXA-AS2 in AML bone marrow tissues.

**Figure 12:**
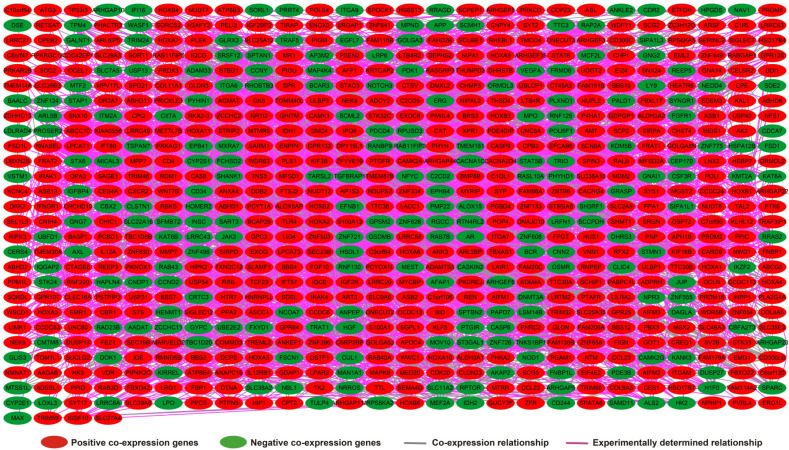
HOXA-AS2-co-expressed PCGs regulatory networks constructed using the STRING online tool.

**Figure 13:**
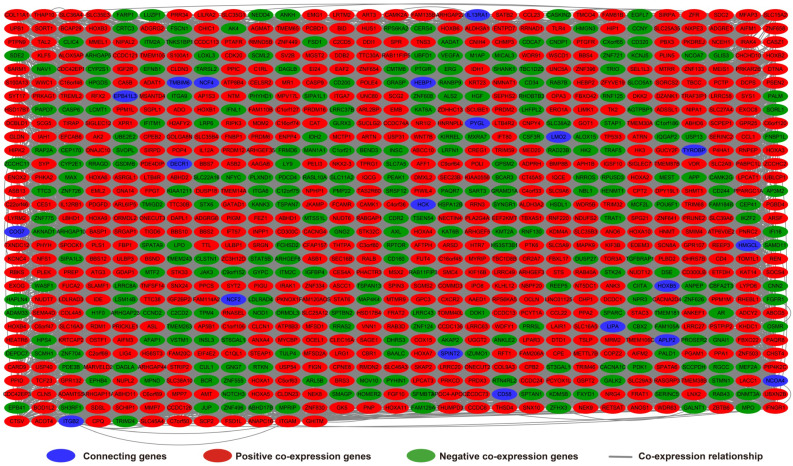
HOXA-AS2-co-expressed PCGs regulatory networks constructed using the GeneMANIA online tool.

**Figure 14:**
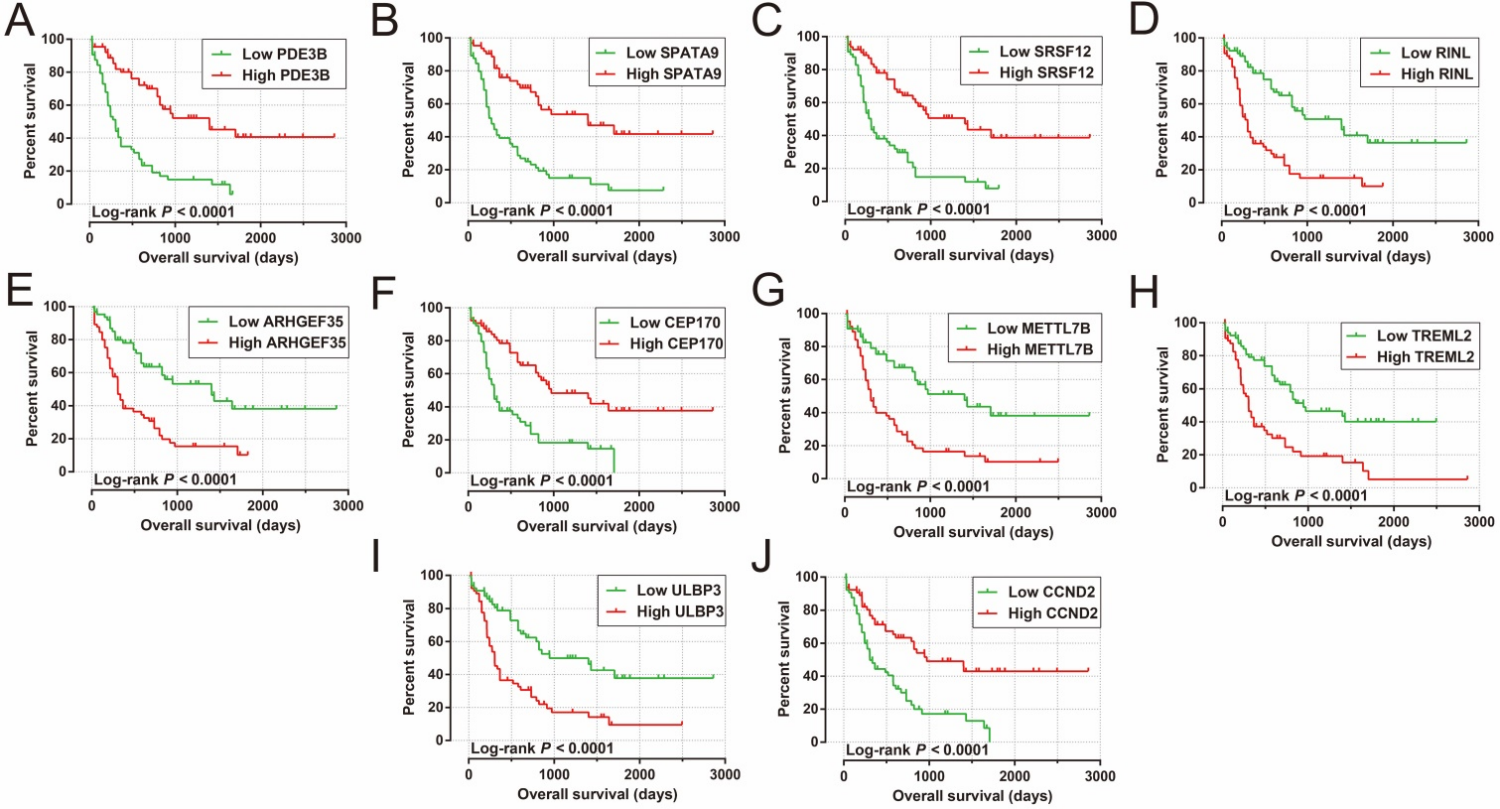
Kaplan-Meier curves of the top ten significantly HOXA-AS2-co-expressed PCGs. (A) *PDE3B*, (B) *SPATA9*, (C) *SRSF12*, (D) *RINL*, (E) *ARHGEF35*, (F) *CEP170*, (G) *METTL7B*, (H) *TREML2*, (I) *ULBP3*, and (J) *CCND2* in AML OS.

Analysis of the PCGs co-expressed with HOXA-AS2 may reflect its function in AML. We detected 903 PCGs co-expressed with HOXA-AS2 in AML bone marrow tissues using the Pearson correlation coefficient for genome-wide co-expression analysis, of which 621 were positively correlated PCGs, while 282 were negatively correlated PCGs (**Figure 11**). All the co-expressed PCGs were significantly associated with HOXA-AS2, with a Pearson correlation coefficient |r|>0.3 and *P*<0.05 (**Table S6**). GO term annotation suggested that these co-expressed PCGs participated in the following biological processes: peroxisome, definitive hemopoiesis, positive regulation of nuclear factor (NF)-κB transcription factor activity, DNA methylation, angiogenesis, intrinsic apoptotic signaling pathway in response to oxidative stress, intracellular signal transduction, Toll-like receptor 4 signaling pathway, and cell migration (**Table S7**). KEGG annotation suggested that these co-expressed PCGs were involved to the peroxisome, Wnt signaling pathway, and pathways in cancer (**Table S8**). BINGO enrichment analysis of these HOXA-AS2-co-expressed PCGs produced no meaningful results. Furthermore, because of the strict set parameters for WGCNA, we were unable to use the HOXA-AS2-co-expressed PCGs to construct a highly connected WGCNA regulatory network. However, we constructed a co-expression regulatory network of these HOXA-AS2-co-expressed PCGs using the STRING and GeneMANIA online tools, which demonstrated the complex interactions between these PCGs. The regulatory networks are shown in **Figures 12** and **13**. Survival analysis of these HOXA-AS2-co-expressed PCGs identified 282 genes that were prominently related to OS in AML patients. The top ten significantly prognostic HOXA-AS2-co-expressed PCGs were as follows: *PDE3B*, spermatogenesis-associated 9 (*SPATA9*), serine and arginine rich splicing factor 12 (*SRSF12*), Ras and Rab interactor-like (*RINL*), *ARHGEF35*, centrosomal protein 170 (*CEP170*), *METTL7B*, triggering receptor expressed on myeloid cells-like 2 (TREML2), *ULBP3*, and cyclin D2 (*CCND2*) (**Figure 14A-J, Table S9**). Four of the top ten significantly prognostic HOXA-AS2-co-expressed PCGs overlapped with the top ten significantly prognostic DEGs: *PDE3B*,* ARHGEF35*, *METTL7B*, and* ULBP3*.

## Discussion

Numerous studies have shown that HOXA-AS2 plays an essential role in cancers and other non-cancer diseases 8, 37. HOXA-AS2 has demonstrated an oncogenic role in a variety of cancers, including pancreatic cancer 38, gastric cancer 39, breast cancer 40, bladder cancer 41, glioma 42, papillary thyroid cancer 43, 44, osteosarcoma 45, non-small lung cancer 46, gallbladder carcinoma 47, colorectal cancer 48, 49, and hepatocellular carcinoma 50, 51. Knockdown of HOXA-AS2 in the above cancer cell lines significantly inhibited tumor cell proliferation, migration, and invasion, and induced cell cycle arrest and apoptosis 38-51, while HOXA-AS2 overexpression promoted cell proliferation in gallbladder carcinoma 47 and osteosarcoma 45. Previous studies also found that HOXA-AS2 was significantly up-regulated in multiple tumor tissues, including pancreatic 38, gastric 39, breast 40, 52, and bladder cancer 41, glioma 42, papillary thyroid cancer 43, 44, osteosarcoma 45, non-small lung cancer 46, gallbladder carcinoma 47, colorectal cancer 48, 49, 53, and hepatocellular carcinoma 50, 51. In our current study, HOXA-AS2 was up-regulated in AML bone marrow tissues by compared with normal bone marrow tissues. Multiple studies have shown that high expression levels of HOXA-AS2 were significantly related to a poor prognosis in patients with gastric cancer 39, breast cancer 40, papillary thyroid cancer 44, non-small lung cancer 46, colorectal cancer 53, and hepatocellular carcinoma 51. Our results also demonstrated that AML patients with high expression of HOXA-AS2 had shorter OS, consistent with the results of previous studies.

Regarding its molecular mechanism in cancers, HOXA-AS2 promoted proliferation and induced epithelial-mesenchymal transition (EMT) via the miR-520c-3p/glypican-3 axis in hepatocellular carcinoma 50, the miR-520c-3p/S100 calcium-binding protein A4 (S100A4) axis in papillary thyroid cancer 43, and the miR-520c-3p/transforming growth factor beta receptor 2/RELA (RELA proto-oncogene, NF-κB subunit) axis in breast cancer^40^. Dong et al. also demonstrated that the HOXA-AS2/miR-520c-3p/S100A4 axis participated in the regulation of adriamycin resistance in AML, and that HOXA-AS2 expression was enhanced in bone marrow tissues of AML patients after treatment with adriamycin-based chemotherapy 9. In addition, HOXA-AS2-induction of EMT through miR-520c-3p was also observed in osteosarcoma 45. HOXA-AS2 also mediated the EMT pathway for oncogene function in colorectal cancer 48 and gallbladder carcinoma 47. Regarding other important molecular mechanisms, HOXA-AS2 promoted cell proliferation through the HOXA-AS2/enhancer of zeste homolog 2 (EZH2)/cyclin-dependent kinase inhibitor 1A (also known as P21), polo-like kinase 3, DNA damage-inducible transcript 3 axis in gastric cancer 39, the HOXA-AS2/EZH2/lysine-specific demethylase 1 (LSD1) in pancreatic cancer 38, and the HOXA-AS2/EZH2 and LSD1/P21 and Kruppel-like factor 2 axis in colorectal cancer 49. Gao et al. suggested that HOXA-AS2 achieved its oncogenic function and angiogenesis in gliomas via the miR-373/epidermal growth factor receptor (EGFR)/VE-cadherin, matrix metallopeptidase 2 (MMP2) and MMP9/PI3K-Akt signaling pathway axis 42. Furthermore, Wang et al. demonstrated that HOXA-AS2 regulated the function of oncogenes in bladder cancer by regulating the HOXA-AS2/miR-125b/SMAD family member 2 axis, and was involved in the regulation of migration, invasion, and stemness of bladder cancer cells. HOXA-AS2 regulated the HOXA-AS2/miR-520a-3p/homeobox D8 and mitogen-activated protein kinase kinase kinase 2 axis in non-small lung cancer 46, and the miR-15a-5p/homeobox A3 (HOXA3) axis in papillary thyroid cancer 44. Zhao et al. reported that HOXA-AS2 regulated the HOXA3/EGFR/Ras/Raf/MAPK kinase/ERK pathway to reduce the sensitivity of glucocorticoids in acute lymphoblastic leukemia 54. In the present study, we performed enrichment analyses of DEGs in AML patients with different expression levels of HOXA-AS2, which also demonstrated that HOXA-AS2 plays an essential role in AML via the PI3K-Akt, ERK, and MAPK pathways regulation, as well as by affecting cell adhesion, cell differentiation, cell migration, angiogenesis, and cell proliferation biological processes. These results are consistent with previous studies. In addition, we also found that the DEGs were enriched in the Wnt, Notch, cell-adhesion molecules, and integrin-mediated signaling pathways, which represent previously unreported associations with HOXA-AS2 regulatory mechanisms. The HOXA-AS2-co-expressed PCGs identified in the present study were enriched in the NF-κB signaling pathway. Relationship between HOXA-AS2 and NF-κB has been reported previously. Zhu et al. reported that HOXA-AS2 was take part in osteogenesis via regulating NF-κB pathway in mesenchymal stem cells 55, and another study found that HOXA-AS2 was involved in inhibiting endothelial inflammation by regulating the NF-κB pathway 37.

Of these three HOXA-AS2-targeted drugs used in AML, carmustine is a broad-spectrum anticancer drug with good curative effect in patients with Hodgkin's disease 56 and AML 57, 58, as well as some curative effect in patients with breast cancer 59, lung cancer 60, brain cancer 61, 62 and brain metastasis 63, 64. Carmustine can be used to treat elderly or comorbid patients with AML, as well as post-transplant acute leukemia in patients with non-Hodgkin's and Hodgkin's lymphoma 57, 58. As is well-known, no studies have revealed the use of cefoxitin for the treatment of AML, but cefoxitin was used to treat colon tumorigenesis induced by the human commensal enterotoxigenic *Bacteroides fragilis* in mice 65. Megestrol acetate can be used to treat advanced endometrial cancer 66, and has also demonstrated an effect in advanced renal cell carcinoma 67-69, and was shown to improve the appetite in patients with advanced cancer 70, 71. However, a review of the literature found no relevant reports of megestrol for AML treatment. Thus, although the present study identified three potential HOXA-AS2-targeted drugs, only carmustine has been reported to show efficacy for the treatment of AML.

This study had some limitations. This was a single-cohort study based on the TCGA dataset, and additional independent cohorts are required to verify our results. Second, although we performed functional enrichment analyses to investigate the potential molecular mechanisms of HOXA-AS2 in AML using multiple bioinformatics tools, *in vivo* and *in vitro* experiments are needed to confirm these findings. Third, the small-molecule HOXA-AS2-targeted drugs screened using the AML whole genome dataset and CMap tool need further experimental verification. Despite these limitations, the present study found that HOXA-AS2 could be a prognostic indicator for AML. In addition, we also used a genome-wide dataset of AML bone marrow tissues to explore the molecular mechanisms of HOXA-AS2 in AML, and screened out three potential small-molecule targeted drugs. These findings thus provide a theoretical basis for further investigations of the functions and mechanisms of HOXA-AS2 in AML.

## Conclusions

Our study provides the first evidence for the use of HOXA-AS2 as a prognostic marker for AML, with high HOXA-AS2 expression related to a poor prognosis in AML patients. Our findings also suggested that HOXA-AS2 levels may be significantly up-regulated in the bone marrow tissues of AML patients compared with healthy subjects. Using multiple bioinformatics analysis tools, we determined the potential molecular mechanisms of HOXA-AS2 in AML, and screened out three small-molecule drugs (megestrol, carmustine, and cefoxitin) as potential HOXA-AS2-targeted therapeutic agents in AML patients. We also identified multiple genes associated with AML prognosis. However, further studies were required to verify these findings.

## Supplementary Material

Supplementary figures.

Supplementary tables.
