# Supplementary material for: Comprehensive investigation of the clinical significance of long non-coding RNA HOXA-AS2 in acute myeloid leukemia using genome-wide RNA sequencing dataset
Source: J Cancer. 2021 Feb 21;12(7):2151–64. doi: 10.7150/jca.48045 (PMC7974522; doi:10.7150/jca.48045)
Supplement: Supplementary file 1 — Supplementary figures. [file jcav12p2151s1.pdf]

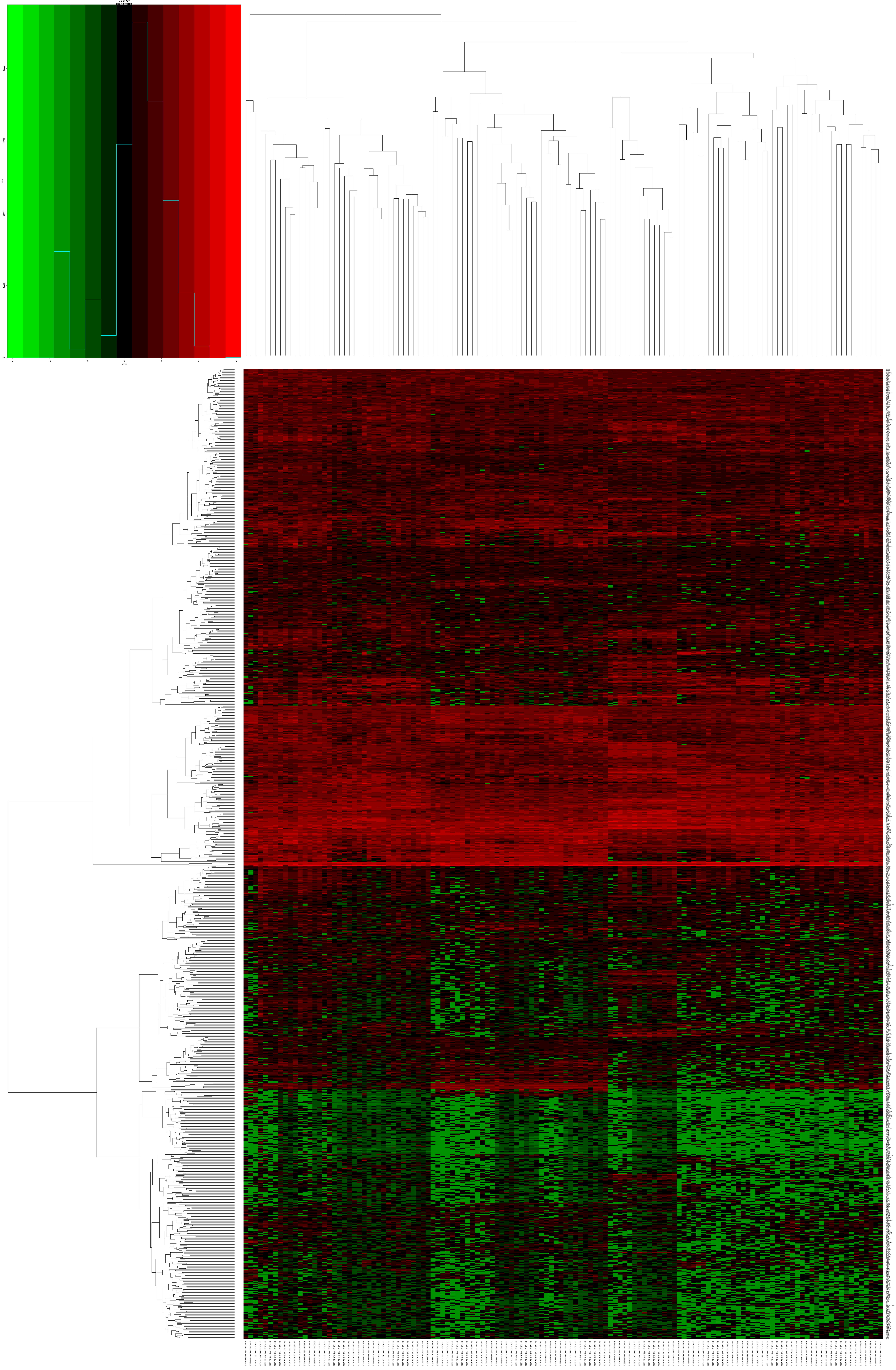

Figure S1. Heat map of DEGs in low- and high-HOXA-AS2-expressing AML patients.

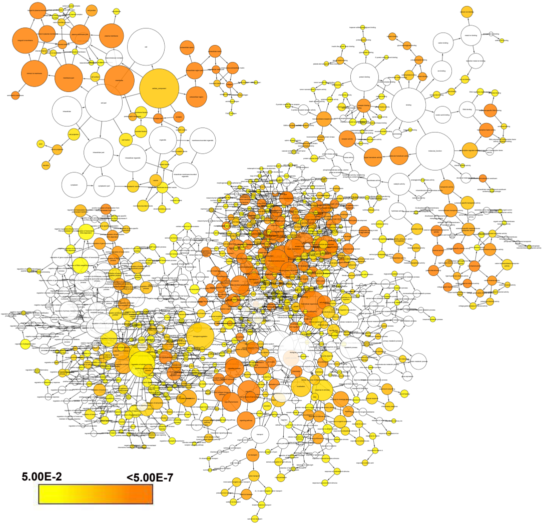

Figure S2. Directed acyclic graph of BiNGO enrichment results of DEGs in low- and high-HOXA-AS2-expressing AML patients.

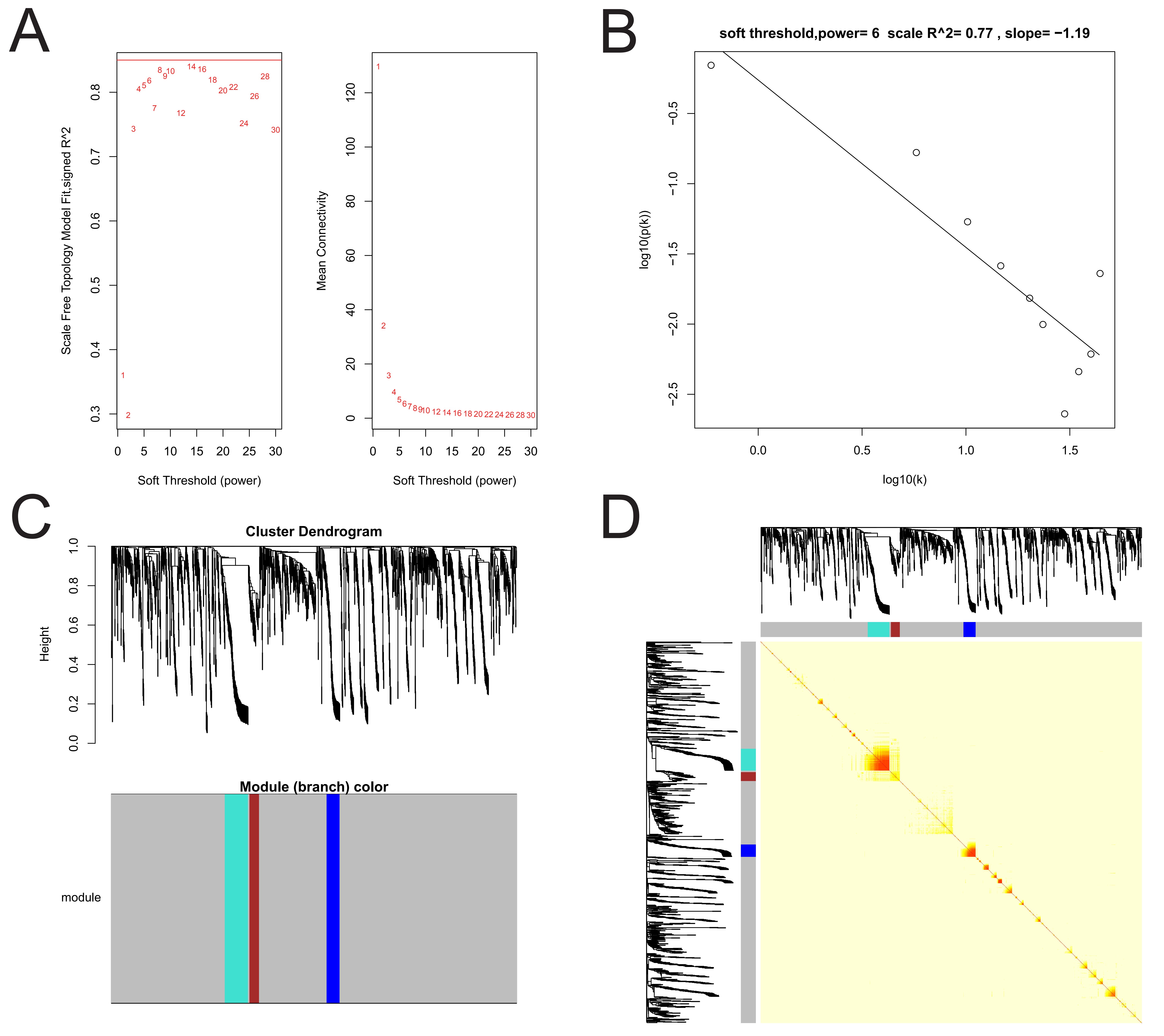

Figure S3. Plot of WGCNA analysis of DEGs in low- and high-HOXA-AS2-expressing AML patients. (A) Soft threshold screening plot; (B) scale-free topology plot; (C) clustering dendrograms of genes; (D) TOM plot.
